# *EZTraits*: A programmable tool to evaluate multi-site deterministic traits

**DOI:** 10.1371/journal.pone.0259327

**Published:** 2022-05-09

**Authors:** Matt Carland, Haley Pedersen, Madhuchanda Bose, Biljana Novković, Charles Manson, Shany Lahan, Alex Pavlenko, Puya G. Yazdi, Manfred G. Grabherr

**Affiliations:** SelfDecode.com, Miami, Florida, United States of America; Al Mansour University College-Baghdad-Iraq, IRAQ

## Abstract

The vast majority of human traits, including many disease phenotypes, are affected by alleles at numerous genomic loci. With a continually increasing set of variants with published clinical disease or biomarker associations, an easy-to-use tool for non-programmers to rapidly screen VCF files for risk alleles is needed. We have developed *EZTraits* as a tool to quickly evaluate genotype data against a set of rules defined by the user. These rules can be defined directly in the scripting language *Lua*, for genotype calls using variant ID (RS number) or chromosomal position. Alternatively, *EZTraits* can parse simple and intuitive text including concepts like ’*any*’ or ’*all*’. Thus, *EZTraits* is designed to support rapid genetic analysis and hypothesis-testing by researchers, regardless of programming experience or technical background. The software is implemented in C++ and compiles and runs on Linux and MacOS. The source code is available under the MIT license from https://github.com/selfdecode/rd-eztraits.

## Introduction

Although many common health disorders are highly polygenic, requiring the calculation of a complex aggregate genetic risk score, there is a subset of traits and disorders for which a few variants with disproportionately large effect sizes account for a significant portion of phenotypic variance. These mono- or oligogenic traits are therefore amenable to simpler analytical approaches, which do not rely on statistical association. Often, these traits can be easily identified with simple analyses and determination of the presence/absence of associated variants.

One illustrative example is the *APOE* gene, in which a two-SNP haplotype may modulate an individual’s risk of late-onset Alzheimer’s disease by approximately 15x [1]. Another example is the ability to digest lactose into adulthood, which can be fully predicted on the basis of just six SNPs in the *MCM6* gene, among which a single heterozygous- or homozygous-derived genotype implies lactose tolerance [2]. Similarly, dietary tolerance to fructose can be predicted by the presence of a few different combinations of homozygous mutations in the *ALDOB* gene [3].

Furthermore, small numbers of variants may also be useful for characterizing individual variability within specific biological pathways. One example is the *COMT* gene, in which various four-SNP haplotypes have been associated with significant differences in the biological activity of the gene’s product enzyme [4, 5]. Even in the absence of a direct link to a clinical phenotype, such genetic markers may serve as a useful “jumping-off” point for further investigations into the etiological structure of clinically relevant phenotypes.

As whole-genome sequencing becomes more routine, many of these traits can be interrogated directly from genomic data. However, a typical sequencing project can produce millions of variants, and parsing through variant files often requires specialized programming knowledge. The difficulty of attracting and training enough researchers with the requisite programming and computing skills is well known [6]. In response, there has been a move towards intuitive GUIs as well as script-based tools free from expensive licenses. These solutions broaden access to the ability to computationally analyze the wealth of genomic data being generated. Indeed, the shortfall in computing skills for data analysis has been recorded in many surveys. Moreover, the situation is not improving, in fact, the skills gap is only set to widen [7]; this makes the development of tools to narrow this gap especially important. At present, the authors are not aware of any open-source, user-friendly programs that are tailored to the analysis of simple, deterministic traits from genomic data.

Here, we present *EZTraits*, a tool specifically designed for non-programmers. *EZTraits* is intended to assist with searching VCF (Variant Call Format [8]) files for the presence of mono- or oligogenic traits and returning their trait associations to the user, based either on our library of variant-trait associations or new, user-added conditions and associations. Thus, *EZTraits* allows genomic researchers to analyze a wide variety of phenotypes of clinical and scientific interest quickly and easily, regardless of their level of programming ability.

## Methods

*EZTraits* evaluates variant combinations by internally building and interpreting *Lua* scripts. The *Lua* programming language [9] was designed with ease of use in mind and has been widely adopted by non-programmers for computer game modding and writing plug-ins, making it a natural choice for use by researchers both with or without a coding background.

There are two ways for users to build analyses with *EZTraits*: (a) by writing or modifying a *Lua* “snippet,” which contains pre-made variables for supplying key genotype and phenotype information; or (b) by writing a plaintext rule set that provides genotype and phenotype information by using simple concepts such as ‘*all*’ and ‘*any’*, which allows for a more intuitive and compact representation. This conversion feature allows users to easily write in rsID-trait associations to use with *EZTraits* without any “coding” at all.

### Using scripts

Users can write *Lua* snippets directly by providing the appropriate genotype and phenotype information. For genotypes, SNPs can be referenced using either their rsID or chromosomal position (following the syntax ‘chr1:6658743’). Phenotype information is entered by modifying two return variables: the floating-point variable ‘*risk*’; and the string ‘*comment*’—both of which can be manipulated directly in the *Lua* script snippet. These two variables allow the user to flexibly provide either quantitative or qualitative phenotype data (or both), depending on the trait being analyzed.

For example, the snippet:

if rs568149713 == "A/G" and rs557514207 == "G/G"then

   comment = "highrisk"

   risk = 0.8

 end

if chr1:16949 == "A/C" or rs553090414 == "C/C" then

   comment = "mediumrisk"

   risk = 0.5

end

is completed into a valid *Lua* function by adding variables that correspond to the RS identifiers or chromosomal positions. These variables are automatically initialized from a VCF or TSV file, and together with a small amount of bracketing code, the complete function is:

function evaluate ()

   comment = "none"

   risk = 0

   rs568149713 = "A/G"

   rs557514207 = "G/G"

   chr1_16949 = "A/C"

   rs553090414 = "C/C"

   if rs568149713 == "A/G" and rs557514207 == "G/G" then

      comment = "highrisk"

      risk = 0.8

   end

   if chr1_16494 == "A/C" or rs553090414 == "C/C" then

      comment = "mediumrisk"

      risk = 0.5

   end

   return risk, comment

end

This function is then called directly from C++ by *EZTraits*, and the results are presented to the user.

### Structured text entry

In addition, *EZTraits* can automatically convert text files into *Lua* by applying some simple-yet-intuitive concepts, such as ‘*any*’ and ‘*all*’, i.e., any of the following conditions satisfy a trait, or all in combination do. This text ruleset is then automatically converted into fully-functional *Lua* code via the tool *Txt2Lua*.

For example, the rules to define fructose tolerance/intolerance using three common causal SNPs can be written as:

Any

rs1800546 ‘GG’

rs76917243 ‘TT’

rs78340951 ‘CC’

== “Fructose Intolerant”

Any:

rs1800546 ‘C/G’

rs76917243 ‘G/T’

rs78340951 C/G

== “Variant Carrier”

else “Tolerant to Fructose”

*EZTraits* accepts and interprets the keywords ‘All’, ‘Any’, and ‘else’, optionally followed by a colon. Acceptable genotype call formats include ‘CG’ and C/G (with optional single quotation marks), where the latter convention has to be used for sites that contain indels, e.g., ‘T/TGAT’.

The above text thus translates into the *Lua* snippet:

if rs1800546 == "G/G" then

   comment = "FructoseIntolerant"

   return risk, comment

end

if rs76917243 == "T/T" then

   comment = "FructoseIntolerant"

   return risk, comment

end

if rs78340951 == "C/C" then

   comment = "FructoseIntolerant"

   return risk, comment

end

if rs1800546 == "C/G" then

   comment = "VariantCarrier"

   return risk, comment

end

if rs76917243 == "G/T" then

   comment = "VariantCarrier"

   return risk, comment

end

if rs78340951 == "C/G" then

   comment = "VariantCarrier"

   return risk, comment

end

comment = "ToleranttoFructose"

## Results

*EZTraits* is a command-line tool that compiles and runs on Linux and Mac operating systems. Inputs are VCF or space/tab-delimited TSV files. The *Lua* interpreter, version 5.4.2, is embedded so that *EZTraits* has no external dependencies. *EZTraits* has minimal requirements in terms of RAM, using less than 5KB on average. It takes about 2.4 minutes to parse a whole-genome VCF file from a single individual from the 1000 Genomes Project [10], containing ~78 million SNPs.

### Usage

*EZTraits* has two input parameters: (a) the VCF or TSV file; and (b) the *Lua* snippet. The usage for processing a VCF and TSV file is:

./EZTraits -i data/sample.vcf -lua scripts/test.lua
./EZTraitsCSV -i data/sample.csv -lua scripts/test.lua

The output is written to the console. To convert structured text, run e.g.:

./Txt2Lua -i scripts/fructose.txt > fruct_test.lua

## Discussion

We developed *EZTraits* to enable researchers, regardless of programming experience, to easily screen genomic data for user-defined combinations of variants that underlie certain phenotypes or disease risks. While we do not have any large-scale scientific studies, in-house experiments show that even biologists with no training in computer programming can use *EZTraits*’ structured text entry feature to set up systems to inquire genomes for specific traits or hypotheses. Our ultimate goal is to make genomic investigations for specific traits as easy as a Google search. We consider elements of natural language processing as an essential part of this endeavor.

While there are many scripts and tools publicly available, we are not aware of any programs to screen VCFs for predefined variant-trait associations that are accessible for the non-programming community. Therefore, to examine these deterministic variants, researchers or medical professionals must either perform targeted genetic testing, or when genomic data is available, use VCF processing tools that require a higher-level of bioinformatics experience, like bcftools [11]. Additionally, interpretation of each genotype call with respect to a given trait is still necessary. This can prove impractical if many individuals or traits are being studied and a researcher lacks programming experience. *EZTraits* provides a simple solution to streamline such workflows.

The most comparable VCF screening tools are designed to facilitate variant discovery or identify causative mutations in patients with Mendelian disorders (e.g., VCF-Miner [12]), BrowseVCF [13], and MendelMD [14]). These tools integrate annotations from functional databases (e.g., ANNOVAR, SnpEFF, Variant Effect Predictor) to try to find mutations that could explain observed phenotypes. Conversely, *EZTraits* is designed to rapidly screen VCFs for known deterministic variants and support hypothesis-testing of suspected variant-trait associations.

Unlike the tools listed above, which are dependent on existing databases, *EZTraits* is flexible and customizable with no external dependencies. Therefore, it is also appropriate for use in non-human and non-model species. For example, within agriculture, genomics has facilitated targeted selection through the identification of causative loci for desirable traits in economically important species [15]. Without genetic insight, selection for profitable phenotypes (e.g., muscular hyperplasia from myostatin mutations in cattle and other livestock) can also lead to the widespread propagation of deleterious mono- or oligogenic disorders if a sire is an unknown carrier of disease-associated variants [16]. The ability to screen sires for carrier status is thus vital for herd health. However, existing tools may not provide the necessary annotations or species-specific resources to do so. *EZTraits* can identify carrier status or provide phenotype predictions in any species because the library of variant-trait associations is user-defined. Additionally, the library can easily be expanded as new associations are identified, with no limit on the number of variants or traits.

Importantly, while *EZTraits* can screen VCFs to help predict phenotypes for simple mono- or oligogenic traits, it does not predict phenotypes or disease risk for complex, polygenic traits, wherein the trait is influenced by a large number of variants each with small effect sizes [17]. More specifically, *EZTraits* is not a statistical tool and does not support the calculation of GWAS-derived polygenic risk scores. One big limitation at present is that *EZTraits* cannot make use of phasing information. However, for certain oligogenic traits, gametic phase (e.g., whether variants are *cis* or *trans* on homologous chromosomes) can affect phenotypic expression [18]. We plan to integrate phase-awareness in future releases, as well as multi-sample VCF processing for even greater efficiency. By design, *EZTraits’* structured language input feature is inclusive and makes it usable for all researchers, including scientists that do not have a background in programming, computer science, or bioinformatics. While “structured language” is just a small step towards a new paradigm of human-machine interaction, genomics is a perfect sandbox for experiments, because very specific questions can be well formulated. In the future, we envision that this approach will be expanded to a more “natural language” interface with a “natural dialog” component, ensuring that all the relevant information and hypotheses are well defined for the machine before starting experiments.
