# Supplementary material for: EZTraits: A programmable tool to evaluate multi-site deterministic traits
Source: PLoS One. 2022 May 9;17(5):e0259327. doi: 10.1371/journal.pone.0259327 (PMC9084532; doi:10.1371/journal.pone.0259327)
Supplement: Attachment — Submitted filename: Detailed_response.docx [file pone.0259327.s001.docx]

**Point-by-point responses**

We thank the authors for their thoughtful comments and valuable suggestions on how to improve the manuscript. Below, you will find our detailed responses point by point.

**Reviewer #1**: The manuscript "EZTraits: a Programmable Tool to Evaluate Multi-site Deterministic Traits" by Matt Carland et al. describes a simple tool for non-programmers to quickly screen VCF files for risk alleles. This paper makes an important contribution to the field because, at the moment, only EZTraits evaluates variant combinations by internally building and interpreting Lua scripts, which is especially useful for non-programmers. While the paper is interesting and presents novel results, I believe a few changes are required before publication:

-Authors should describe the benefits and drawbacks of using EZtraits, as well as its limitations in comparison to other similar software.

Response: thank you, we added a discussion section in which we detail the scenarios in which EZTraits is useful, and scenarios in which it is not.

**Reviewer #2**: Carland et al describes their software (EZTraits) to evaluate complex traits using genotype data from microarrays. This is a pure software description.

The authors should evaluate their software in different data sets and show its performance in for those data sets using appropriate plots and statistics. They should also compare the performance of their software with other available softwares and discuss its pitfalls and strengths in the discussion section.

Response: thank you, we clarified the test to better define the exact problem that EZTraits solves. We also added a discussion section in which we point out the differences to other tools, which address different problems

**Reviewer #3**: This manuscript is short and sweet. A bit short at times, but I chose to accept it anyway based on the purpose it presents. The time and age is totally here to democratize genetics and make the analysis tools more readably available to an upcoming generation of researchers, many with limited coding abilities. Even in a Life Science “advanced” country like Sweden we are severely lacking clinical geneticists and genetic counselors as a profession. We are as a nation producing excellent Bioinformaticians, but the education system is lengthy and not updated to current needs, so tools like EZTraits presents a very attractive solution to this problem, which would empower our biomedical community with tools to address the ever-growing mountains of data we are producing. Genomics is here to stay with unprecedented ability in lower costs and producing medical data, that needs to be interpreted in a streamlined fashion.

Response: thank you for your kind words, and we agree wholeheartedly!

General:
The text parts are a bit weak. They could spend a bit more time elaborating on the introduction section and there is no conclusion section at all.

Response: agreed, we clarified the introduction and added a discussion section (see below).

Authors provide source code which is great, and it looks good.


Specific comments
1) In the abstract row 5, they should avoid giving the example of microarrays. NGS and other methods are starting to dominate this industry, so why limit scope and applications use to microarray, which might make some potential reader less excited about the content.

Response: thank you for catching that, we fixed it!

2) In the introduction section, they give some nice examples of interesting SNPs and the complications of polygenic risk scores. However they do not give us readers a bigger overview of how many variants there really is in a VCF file, say from a WGS sequencing run. They could make the whole manuscript a bit more interesting by including a small section discussing this diversity between various individuals and why this tool they are presenting provides a very attractive solution to analyze it.

Response: good point, we added some numbers to the results section to highlight that even for millions of SNPs, the tool only takes a few minutes.

3) The introduction or (possibly the results/conclusion section) does not provide comparison to currently available tools and where is stands against them. Would love to get some pros and cons.

Response: agreed, we now discuss the scope of EZTraits versus the scope of other tools in detail in the newly added discussion section.

4) Polygenic risk score (PRS) is a risky task. They don’t specify challenges of this tool for users.

Response: thank you for pointing out that the phrasing was misleading, we edited the text. PRS is indeed risky business, because results from one cohort or population is not guaranteed. Our tool, on the other hand, targets phenotypes or diseases for which the mechanisms are known – or at least better understood - albeit they can be poly-genic or based on haplotypes rather than individual SNPs.

5) Also since their mission is to address the non-programmers market segment, they could also include a section in the introduction that addresses this issue. E.g. provide figures on how many researchers would be potential users today, but cannot since they lack programming skills, and how many backlogged bioinformaticians there are. Since the paper is coming from a company, they should easily be able to pull up this time of marketing material, which is important even here with a scientific audience to pinpoint the reason for the tool. Simply phrased, highlight what the contribution of this tool is for scientific community?

Response: while we appreciate your suggestion, we strongly prefer not to use any data from our marketing department, since we feel that whatever numbers they give us are way overly optimistic.

6) The Results section is well-written and no comments but could include parts from comment 4.

7) I would have loved to see a conclusion section detailing the future of this and highlighting the tools capabilities a bit more.

Response: this is a great suggestion, thank you! We added a Discussion section that explains the tool’s capabilities and what specific problem it solves.
