# Supplementary material for: EZTraits: A programmable tool to evaluate multi-site deterministic traits
Source: PLoS One. 2022 May 9;17(5):e0259327. doi: 10.1371/journal.pone.0259327 (PMC9084532; doi:10.1371/journal.pone.0259327)
Supplement: Attachment — Submitted filename: Point-by-point.docx [file pone.0259327.s002.docx]

### Reviewer comments and point-by-point responses

We again thank all the reviewers for their thoughtful comments and valuable suggestions. We fixed all the remaining issues, please see for detailed responses below.

**Reviewer #1**: The manuscript titled "EZTraits: a Programmable Tool to Evaluate Multi-site Deterministic Traits" has some merit and falls within the scope of PLOS One. However, the authors are yet to address the issues raised in the previous submission. The authors did not provide clear/well-organized discussion and conclusion sections. The discussion should NOT be a part of the introduction section, and to make the discussion section engaging; the authors should include the following information:

-The major findings of your study

Response: we clearly highlight this in the discussion now. We realize that we did not conduct large scale usability studies, yet the overwhelming positive response from geneticists and biologists in our organization – most of which did not even know that they were writing code that was executed by a computer verbatim – leads us to expect that this approach to human-machine interfaces will benefit research in many areas, and will ultimately bridge the gap between (even vehement) non-programmers and computers. Our ultimate goal is to make phenotype and trait queries – along with hypothesis testing and verification – as simple as a Google search.

-How these findings relate to what others have done

Response: we added some text to the discussion section; in short, there are two common responses: (a) throw experimental evidence at the problem, such as PCR; or (b) hire a bioinformatics student, Post-Doc, etc. to mine this information from already existing data. Problem with point 1 is that these experiments will fail in capturing combinatorial effects; and (b) frankly, what do you do with the bioinformatician once she or he ran bcftools, did some more scripting, did the analysis? This is the way many researchers in biology and medicine think, so these bioinformaticians never get hired.

I do have to point out that Sweden - also Switzerland, and some institutions in the US - have found ingenious solutions to this problem, yet here we are, offering an alternative, which will by no means replace these efforts. We just lower the bar for non-programmers.

-Limitations of your findings

Response: We added a sentence about the limitations to the discussion. The biggest shortcoming of EZTraits is that it is not aware of phasing, hence haplotype-dependent traits elude the scope of the program as it is. However, this was a conscious decision taken by the developers of EZTraits to defer that feature until we are certain that the user will not inadvertently enter incorrect statements or code.

-An explanation for any surprising, unexpected, or inconclusive results

Response: yeah, we were surprised to see biologists and medical researchers writing computer code – without them even knowing it. That is difficult to quantify though, so we will refer to future research, in that...

-Suggestions for further research

Response: we added some text at the end of the discussion. Our ultimate goal is that also computer-phobe researchers will have direct access to complex analyses. That might sound a bit Utopian, yet it is achievable. We start with structured language – which is a step up from programming language – will inch our way into more natural language, which implies that we have a real dialog with the machine, and will end up with something as easy to use as a smartphone. We have to take one step at a time, and EZTraits is a test balloon in this journey.
