# Supplementary material for: EZTraits: A programmable tool to evaluate multi-site deterministic traits
Source: PLoS One. 2022 May 9;17(5):e0259327. doi: 10.1371/journal.pone.0259327 (PMC9084532; doi:10.1371/journal.pone.0259327)
Supplement: Peer review history

# Peer review: Decision Letter 0

**DOI:** 10.1371/journal.pone.0259327.r001

**Authors:** Ahmed Mancy Mosa

13 Dec 2021

PONE-D-21-32657EZTraits: a Programmable Tool to Evaluate Multi-site Deterministic TraitsPLOS ONE

Dear Dr. Grabherr,

Thank you for submitting your manuscript to PLOS ONE. After careful consideration, we feel that it has merit but does not fully meet PLOS ONE’s publication criteria as it currently stands. Therefore, we invite you to submit a revised version of the manuscript that addresses the points raised during the review process.

Please consider all comments of all reviewers including reviewer 2

Please submit your revised manuscript by Jan 27 2022 11:59PM. If you will need more time than this to complete your revisions, please reply to this message or contact the journal office at plosone@plos.org. When you're ready to submit your revision, log on to https://www.editorialmanager.com/pone/ and select the 'Submissions Needing Revision' folder to locate your manuscript file.

Please include the following items when submitting your revised manuscript:

- A rebuttal letter that responds to each point raised by the academic editor and reviewer(s). You should upload this letter as a separate file labeled 'Response to Reviewers'.
- A marked-up copy of your manuscript that highlights changes made to the original version. You should upload this as a separate file labeled 'Revised Manuscript with Track Changes'.
- An unmarked version of your revised paper without tracked changes. You should upload this as a separate file labeled 'Manuscript'.

If you would like to make changes to your financial disclosure, please include your updated statement in your cover letter. Guidelines for resubmitting your figure files are available below the reviewer comments at the end of this letter.

If applicable, we recommend that you deposit your laboratory protocols in protocols.io to enhance the reproducibility of your results. Protocols.io assigns your protocol its own identifier (DOI) so that it can be cited independently in the future. For instructions see: https://journals.plos.org/plosone/s/submission-guidelines#loc-laboratory-protocols. Additionally, PLOS ONE offers an option for publishing peer-reviewed Lab Protocol articles, which describe protocols hosted on protocols.io. Read more information on sharing protocols at https://plos.org/protocols?utm_medium=editorial-email&utm_source=authorletters&utm_campaign=protocols.

We look forward to receiving your revised manuscript.

Kind regards,

Ahmed Mancy Mosa, Ph.D.

Academic Editor

PLOS ONE

Journal Requirements:

When submitting your revision, we need you to address these additional requirements.

1. Please ensure that your manuscript meets PLOS ONE's style requirements, including those for file naming. The PLOS ONE style templates can be found at

https://journals.plos.org/plosone/s/file?id=wjVg/PLOSOne_formatting_sample_main_body.pdf and

https://journals.plos.org/plosone/s/file?id=ba62/PLOSOne_formatting_sample_title_authors_affiliations.pdf

2. We note that the grant information you provided in the ‘Funding Information’ and ‘Financial Disclosure’ sections do not match.

When you resubmit, please ensure that you provide the correct grant numbers for the awards you received for your study in the ‘Funding Information’ section.

3. Thank you for stating the following in the Competing Interests section:

“All authors are either employed by and/or hold stock or stock options in SelfDecode. In addition, PGY has equity in Systomic Health LLC and Ethobiotics LLC. There are no other relevant activities or financial relationships which have influenced this work.”

Please confirm that this does not alter your adherence to all PLOS ONE policies on sharing data and materials, by including the following statement: "This does not alter our adherence to  PLOS ONE policies on sharing data and materials.” (as detailed online in our guide for authors http://journals.plos.org/plosone/s/competing-interests).  If there are restrictions on sharing of data and/or materials, please state these. Please note that we cannot proceed with consideration of your article until this information has been declared.

Please include your updated Competing Interests statement in your cover letter; we will change the online submission form on your behalf.

[Note: HTML markup is below. Please do not edit.]

Reviewers' comments:

Reviewer's Responses to Questions

**Comments to the Author**

1. Is the manuscript technically sound, and do the data support the conclusions?

The manuscript must describe a technically sound piece of scientific research with data that supports the conclusions. Experiments must have been conducted rigorously, with appropriate controls, replication, and sample sizes. The conclusions must be drawn appropriately based on the data presented. 

Reviewer #1: Yes

Reviewer #2: No

Reviewer #3: Yes

**********

2. Has the statistical analysis been performed appropriately and rigorously? 

Reviewer #1: Yes

Reviewer #2: No

Reviewer #3: N/A

**********

3. Have the authors made all data underlying the findings in their manuscript fully available?

The PLOS Data policy requires authors to make all data underlying the findings described in their manuscript fully available without restriction, with rare exception (please refer to the Data Availability Statement in the manuscript PDF file). The data should be provided as part of the manuscript or its supporting information, or deposited to a public repository. For example, in addition to summary statistics, the data points behind means, medians and variance measures should be available. If there are restrictions on publicly sharing data—e.g. participant privacy or use of data from a third party—those must be specified.

Reviewer #1: Yes

Reviewer #2: No

Reviewer #3: Yes

**********

4. Is the manuscript presented in an intelligible fashion and written in standard English?

PLOS ONE does not copyedit accepted manuscripts, so the language in submitted articles must be clear, correct, and unambiguous. Any typographical or grammatical errors should be corrected at revision, so please note any specific errors here.

Reviewer #1: Yes

Reviewer #2: Yes

Reviewer #3: Yes

**********

5. Review Comments to the Author

Please use the space provided to explain your answers to the questions above. You may also include additional comments for the author, including concerns about dual publication, research ethics, or publication ethics. (Please upload your review as an attachment if it exceeds 20,000 characters)

Reviewer #1: The manuscript "EZTraits: a Programmable Tool to Evaluate Multi-site Deterministic Traits" by Matt Carland et al. describes a simple tool for non-programmers to quickly screen VCF files for risk alleles. This paper makes an important contribution to the field because, at the moment, only EZTraits evaluates variant combinations by internally building and interpreting Lua scripts, which is especially useful for non-programmers. While the paper is interesting and presents novel results, I believe a few changes are required before publication:

-Authors should describe the benefits and drawbacks of using EZtraits, as well as its limitations in comparison to other similar software.

Reviewer #2: Carland et al describes their software (EZTraits) to evaluate complex traits using genotype data from microarrays. This is a pure software description.

The authors should evaluate their software in different data sets and show its performance in for those data sets using appropriate plots and statistics. They should also compare the performance of their software with other available softwares and discuss its pitfalls and strengths in the discussion section.

Reviewer #3: This manuscript is short and sweet. A bit short at times, but I chose to accept it anyway based on the purpose it presents. The time and age is totally here to democratize genetics and make the analysis tools more readably available to an upcoming generation of researchers, many with limited coding abilities. Even in a Life Science “advanced” country like Sweden we are severely lacking clinical geneticists and genetic counselors as a profession. We are as a nation producing excellent Bioinformaticians, but the education system is lengthy and not updated to current needs, so tools like EZTraits presents a very attractive solution to this problem, which would empower our biomedical community with tools to address the ever-growing mountains of data we are producing. Genomics is here to stay with unprecedented ability in lower costs and producing medical data, that needs to be interpreted in a streamlined fashion.

General:

The text parts are a bit weak. They could spend a bit more time elaborating on the introduction section and there is no conclusion section at all.

Authors provide source code which is great, and it looks good.

Specific comments

1) In the abstract row 5, they should avoid giving the example of microarrays. NGS and other methods are starting to dominate this industry, so why limit scope and applications use to microarray, which might make some potential reader less excited about the content.

2) In the introduction section, they give some nice examples of interesting SNPs and the complications of polygenic risk scores. However they do not give us readers a bigger overview of how many variants there really is in a VCF file, say from a WGS sequencing run. They could make the whole manuscript a bit more interesting by including a small section discussing this diversity between various individuals and why this tool they are presenting provides a very attractive solution to analyze it.

3) The introduction or (possibly the results/conclusion section) does not provide comparison to currently available tools and where is stands against them. Would love to get some pros and cons.

4) Polygenic risk score (PRS) is a risky task. They don’t specify challenges of this tool for users.

5) Also since their mission is to address the non-programmers market segment, they could also include a section in the introduction that addresses this issue. E.g. provide figures on how many researchers would be potential users today, but cannot since they lack programming skills, and how many backlogged bioinformaticians there are. Since the paper is coming from a company, they should easily be able to pull up this time of marketing material, which is important even here with a scientific audience to pinpoint the reason for the tool. Simply phrased, highlight what the contribution of this tool is for scientific community?

6) The Results section is well-written and no comments but could include parts from comment 4.

7) I would have loved to see a conclusion section detailing the future of this and highlighting the tools capabilities a bit more.

**********

6. PLOS authors have the option to publish the peer review history of their article (what does this mean?). If published, this will include your full peer review and any attached files.

If you choose “no”, your identity will remain anonymous but your review may still be made public.

**Do you want your identity to be public for this peer review?** For information about this choice, including consent withdrawal, please see our Privacy Policy.

Reviewer #1: No

Reviewer #2: No

Reviewer #3: No

[NOTE: If reviewer comments were submitted as an attachment file, they will be attached to this email and accessible via the submission site. Please log into your account, locate the manuscript record, and check for the action link "View Attachments". If this link does not appear, there are no attachment files.]

While revising your submission, please upload your figure files to the Preflight Analysis and Conversion Engine (PACE) digital diagnostic tool, https://pacev2.apexcovantage.com/. PACE helps ensure that figures meet PLOS requirements. To use PACE, you must first register as a user. Registration is free. Then, login and navigate to the UPLOAD tab, where you will find detailed instructions on how to use the tool. If you encounter any issues or have any questions when using PACE, please email PLOS at figures@plos.org. Please note that Supporting Information files do not need this step.

# Author comment: Author response to Decision Letter 0

**DOI:** 10.1371/journal.pone.0259327.r002

3 Feb 2022

We thank the authors for their thoughtful comments and valuable suggestions on how to improve the manuscript. Below, you will find our detailed responses point by point.

Reviewer #1: The manuscript "EZTraits: a Programmable Tool to Evaluate Multi-site Deterministic Traits" by Matt Carland et al. describes a simple tool for non-programmers to quickly screen VCF files for risk alleles. This paper makes an important contribution to the field because, at the moment, only EZTraits evaluates variant combinations by internally building and interpreting Lua scripts, which is especially useful for non-programmers. While the paper is interesting and presents novel results, I believe a few changes are required before publication:

-Authors should describe the benefits and drawbacks of using EZtraits, as well as its limitations in comparison to other similar software.

Response: thank you, we added a discussion section in which we detail the scenarios in which EZTraits is useful, and scenarios in which it is not. 

Reviewer #2: Carland et al describes their software (EZTraits) to evaluate complex traits using genotype data from microarrays. This is a pure software description.

The authors should evaluate their software in different data sets and show its performance in for those data sets using appropriate plots and statistics. They should also compare the performance of their software with other available softwares and discuss its pitfalls and strengths in the discussion section.

Response: thank you, we clarified the test to better define the exact problem that EZTraits solves. We also added a discussion section in which we point out the differences to other tools, which address different problems 

Reviewer #3: This manuscript is short and sweet. A bit short at times, but I chose to accept it anyway based on the purpose it presents. The time and age is totally here to democratize genetics and make the analysis tools more readably available to an upcoming generation of researchers, many with limited coding abilities. Even in a Life Science “advanced” country like Sweden we are severely lacking clinical geneticists and genetic counselors as a profession. We are as a nation producing excellent Bioinformaticians, but the education system is lengthy and not updated to current needs, so tools like EZTraits presents a very attractive solution to this problem, which would empower our biomedical community with tools to address the ever-growing mountains of data we are producing. Genomics is here to stay with unprecedented ability in lower costs and producing medical data, that needs to be interpreted in a streamlined fashion.

Response: thank you for your kind words, and we agree wholeheartedly! 

General:

The text parts are a bit weak. They could spend a bit more time elaborating on the introduction section and there is no conclusion section at all.

Response: agreed, we clarified the introduction and added a discussion section (see below). 

Authors provide source code which is great, and it looks good.

Specific comments

1) In the abstract row 5, they should avoid giving the example of microarrays. NGS and other methods are starting to dominate this industry, so why limit scope and applications use to microarray, which might make some potential reader less excited about the content.

Response: thank you for catching that, we fixed it! 

2) In the introduction section, they give some nice examples of interesting SNPs and the complications of polygenic risk scores. However they do not give us readers a bigger overview of how many variants there really is in a VCF file, say from a WGS sequencing run. They could make the whole manuscript a bit more interesting by including a small section discussing this diversity between various individuals and why this tool they are presenting provides a very attractive solution to analyze it.

Response: good point, we added some numbers to the results section to highlight that even for millions of SNPs, the tool only takes a few minutes.

3) The introduction or (possibly the results/conclusion section) does not provide comparison to currently available tools and where is stands against them. Would love to get some pros and cons.

Response: agreed, we now discuss the scope of EZTraits versus the scope of other tools in detail in the newly added discussion section.

4) Polygenic risk score (PRS) is a risky task. They don’t specify challenges of this tool for users.

Response: thank you for pointing out that the phrasing was misleading, we edited the text. PRS is indeed risky business, because results from one cohort or population is not guaranteed. Our tool, on the other hand, targets phenotypes or diseases for which the mechanisms are known – or at least better understood - albeit they can be poly-genic or based on haplotypes rather than individual SNPs.

5) Also since their mission is to address the non-programmers market segment, they could also include a section in the introduction that addresses this issue. E.g. provide figures on how many researchers would be potential users today, but cannot since they lack programming skills, and how many backlogged bioinformaticians there are. Since the paper is coming from a company, they should easily be able to pull up this time of marketing material, which is important even here with a scientific audience to pinpoint the reason for the tool. Simply phrased, highlight what the contribution of this tool is for scientific community?

Response: while we appreciate your suggestion, we strongly prefer not to use any data from our marketing department, since we feel that whatever numbers they give us are way overly optimistic.

6) The Results section is well-written and no comments but could include parts from comment 4.

7) I would have loved to see a conclusion section detailing the future of this and highlighting the tools capabilities a bit more.

Response: this is a great suggestion, thank you! We added a Discussion section that explains the tool’s capabilities and what specific problem it solves.

Attachment Submitted filename: Detailed_response.docx Click here for additional data file.

# Peer review: Decision Letter 1

**DOI:** 10.1371/journal.pone.0259327.r003

**Authors:** Ahmed Mancy Mosa

28 Feb 2022

PONE-D-21-32657R1EZTraits: a Programmable Tool to Evaluate Multi-site Deterministic TraitsPLOS ONE

Dear Dr. Grabherr,

Thank you for submitting your manuscript to PLOS ONE. After careful consideration, we feel that it has merit but does not fully meet PLOS ONE’s publication criteria as it currently stands. Therefore, we invite you to submit a revised version of the manuscript that addresses the points raised during the review process.

Please, carefully, consider all comments

Please submit your revised manuscript by Apr 14 2022 11:59PM. If you will need more time than this to complete your revisions, please reply to this message or contact the journal office at plosone@plos.org. When you're ready to submit your revision, log on to https://www.editorialmanager.com/pone/ and select the 'Submissions Needing Revision' folder to locate your manuscript file.

Please include the following items when submitting your revised manuscript:

- A rebuttal letter that responds to each point raised by the academic editor and reviewer(s). You should upload this letter as a separate file labeled 'Response to Reviewers'.
- A marked-up copy of your manuscript that highlights changes made to the original version. You should upload this as a separate file labeled 'Revised Manuscript with Track Changes'.
- An unmarked version of your revised paper without tracked changes. You should upload this as a separate file labeled 'Manuscript'.

If you would like to make changes to your financial disclosure, please include your updated statement in your cover letter. Guidelines for resubmitting your figure files are available below the reviewer comments at the end of this letter.

If applicable, we recommend that you deposit your laboratory protocols in protocols.io to enhance the reproducibility of your results. Protocols.io assigns your protocol its own identifier (DOI) so that it can be cited independently in the future. For instructions see: https://journals.plos.org/plosone/s/submission-guidelines#loc-laboratory-protocols. Additionally, PLOS ONE offers an option for publishing peer-reviewed Lab Protocol articles, which describe protocols hosted on protocols.io. Read more information on sharing protocols at https://plos.org/protocols?utm_medium=editorial-email&utm_source=authorletters&utm_campaign=protocols.

We look forward to receiving your revised manuscript.

Kind regards,

Ahmed Mancy Mosa, Ph.D.

Academic Editor

PLOS ONE

Journal Requirements:

Please review your reference list to ensure that it is complete and correct. If you have cited papers that have been retracted, please include the rationale for doing so in the manuscript text, or remove these references and replace them with relevant current references. Any changes to the reference list should be mentioned in the rebuttal letter that accompanies your revised manuscript. If you need to cite a retracted article, indicate the article’s retracted status in the References list and also include a citation and full reference for the retraction notice.

[Note: HTML markup is below. Please do not edit.]

Reviewers' comments:

Reviewer's Responses to Questions

**Comments to the Author**

1. If the authors have adequately addressed your comments raised in a previous round of review and you feel that this manuscript is now acceptable for publication, you may indicate that here to bypass the “Comments to the Author” section, enter your conflict of interest statement in the “Confidential to Editor” section, and submit your "Accept" recommendation.

Reviewer #1: (No Response)

Reviewer #3: All comments have been addressed

**********

2. Is the manuscript technically sound, and do the data support the conclusions?

The manuscript must describe a technically sound piece of scientific research with data that supports the conclusions. Experiments must have been conducted rigorously, with appropriate controls, replication, and sample sizes. The conclusions must be drawn appropriately based on the data presented. 

Reviewer #1: Partly

Reviewer #3: Yes

**********

3. Has the statistical analysis been performed appropriately and rigorously? 

Reviewer #1: N/A

Reviewer #3: N/A

**********

4. Have the authors made all data underlying the findings in their manuscript fully available?

The PLOS Data policy requires authors to make all data underlying the findings described in their manuscript fully available without restriction, with rare exception (please refer to the Data Availability Statement in the manuscript PDF file). The data should be provided as part of the manuscript or its supporting information, or deposited to a public repository. For example, in addition to summary statistics, the data points behind means, medians and variance measures should be available. If there are restrictions on publicly sharing data—e.g. participant privacy or use of data from a third party—those must be specified.

Reviewer #1: Yes

Reviewer #3: Yes

**********

5. Is the manuscript presented in an intelligible fashion and written in standard English?

PLOS ONE does not copyedit accepted manuscripts, so the language in submitted articles must be clear, correct, and unambiguous. Any typographical or grammatical errors should be corrected at revision, so please note any specific errors here.

Reviewer #1: (No Response)

Reviewer #3: Yes

**********

6. Review Comments to the Author

Please use the space provided to explain your answers to the questions above. You may also include additional comments for the author, including concerns about dual publication, research ethics, or publication ethics. (Please upload your review as an attachment if it exceeds 20,000 characters)

Reviewer #1: The manuscript titled "EZTraits: a Programmable Tool to Evaluate Multi-site Deterministic Traits" has some merit and falls within the scope of PLOS One. However, the authors are yet to address the issues raised in the previous submission. The authors did not provide clear/well-organized discussion and conclusion sections. The discussion should NOT be a part of the introduction section, and to make the discussion section engaging; the authors should include the following information:

-The major findings of your study

-How these findings relate to what others have done

-Limitations of your findings

-An explanation for any surprising, unexpected, or inconclusive results

-Suggestions for further research

Reviewer #3: Thanks for addressing the review comments in a very smart, efficient, and correct manor. The manuscript has matured a lot, gives more background, scope and utilisation of the important tool that has been described and presented here. The extended introduction and new discussion section add huge value to the manuscript, and it was both educational and enjoyable to read it. Looking forward to downloading the pdf and reading post-publication date.

**********

7. PLOS authors have the option to publish the peer review history of their article (what does this mean?). If published, this will include your full peer review and any attached files.

If you choose “no”, your identity will remain anonymous but your review may still be made public.

**Do you want your identity to be public for this peer review?** For information about this choice, including consent withdrawal, please see our Privacy Policy.

Reviewer #1: No

Reviewer #3: No

[NOTE: If reviewer comments were submitted as an attachment file, they will be attached to this email and accessible via the submission site. Please log into your account, locate the manuscript record, and check for the action link "View Attachments". If this link does not appear, there are no attachment files.]

While revising your submission, please upload your figure files to the Preflight Analysis and Conversion Engine (PACE) digital diagnostic tool, https://pacev2.apexcovantage.com/. PACE helps ensure that figures meet PLOS requirements. To use PACE, you must first register as a user. Registration is free. Then, login and navigate to the UPLOAD tab, where you will find detailed instructions on how to use the tool. If you encounter any issues or have any questions when using PACE, please email PLOS at figures@plos.org. Please note that Supporting Information files do not need this step.

# Author comment: Author response to Decision Letter 1

**DOI:** 10.1371/journal.pone.0259327.r004

14 Apr 2022

Reviewer comments and point-by-point responses

We again thank all the reviewers for their thoughtful comments and valuable suggestions. We fixed all the remaining issues, please see for detailed responses below.

Reviewer #1: The manuscript titled "EZTraits: a Programmable Tool to Evaluate Multi-site Deterministic Traits" has some merit and falls within the scope of PLOS One. However, the authors are yet to address the issues raised in the previous submission. The authors did not provide clear/well-organized discussion and conclusion sections. The discussion should NOT be a part of the introduction section, and to make the discussion section engaging; the authors should include the following information:

-The major findings of your study

Response: we clearly highlight this in the discussion now. We realize that we did not conduct large scale usability studies, yet the overwhelming positive response from geneticists and biologists in our organization – most of which did not even know that they were writing code that was executed by a computer verbatim – leads us to expect that this approach to human-machine interfaces will benefit research in many areas, and will ultimately bridge the gap between (even vehement) non-programmers and computers. Our ultimate goal is to make phenotype and trait queries – along with hypothesis testing and verification – as simple as a Google search.

-How these findings relate to what others have done

Response: we added some text to the discussion section; in short, there are two common responses: (a) throw experimental evidence at the problem, such as PCR; or (b) hire a bioinformatics student, Post-Doc, etc. to mine this information from already existing data. Problem with point 1 is that these experiments will fail in capturing combinatorial effects; and (b) frankly, what do you do with the bioinformatician once she or he ran bcftools, did some more scripting, did the analysis? This is the way many researchers in biology and medicine think, so these bioinformaticians never get hired. 

I do have to point out that Sweden - also Switzerland, and some institutions in the US - have found ingenious solutions to this problem, yet here we are, offering an alternative, which will by no means replace these efforts. We just lower the bar for non-programmers.

-Limitations of your findings

Response: We added a sentence about the limitations to the discussion. The biggest shortcoming of EZTraits is that it is not aware of phasing, hence haplotype-dependent traits elude the scope of the program as it is. However, this was a conscious decision taken by the developers of EZTraits to defer that feature until we are certain that the user will not inadvertently enter incorrect statements or code.

-An explanation for any surprising, unexpected, or inconclusive results

Response: yeah, we were surprised to see biologists and medical researchers writing computer code – without them even knowing it. That is difficult to quantify though, so we will refer to future research, in that...

-Suggestions for further research

Response: we added some text at the end of the discussion. Our ultimate goal is that also computer-phobe researchers will have direct access to complex analyses. That might sound a bit Utopian, yet it is achievable. We start with structured language – which is a step up from programming language – will inch our way into more natural language, which implies that we have a real dialog with the machine, and will end up with something as easy to use as a smartphone. We have to take one step at a time, and EZTraits is a test balloon in this journey.

Attachment Submitted filename: Point-by-point.docx Click here for additional data file.

# Peer review: Decision Letter 2

**DOI:** 10.1371/journal.pone.0259327.r005

**Authors:** Ahmed Mancy Mosa

25 Apr 2022

EZTraits: a Programmable Tool to Evaluate Multi-site Deterministic Traits

PONE-D-21-32657R2

Dear Dr. Grabherr,

We’re pleased to inform you that your manuscript has been judged scientifically suitable for publication and will be formally accepted for publication once it meets all outstanding technical requirements.

Within one week, you’ll receive an e-mail detailing the required amendments. When these have been addressed, you’ll receive a formal acceptance letter and your manuscript will be scheduled for publication.

An invoice for payment will follow shortly after the formal acceptance. To ensure an efficient process, please log into Editorial Manager at http://www.editorialmanager.com/pone/, click the 'Update My Information' link at the top of the page, and double check that your user information is up-to-date. If you have any billing related questions, please contact our Author Billing department directly at authorbilling@plos.org.

If your institution or institutions have a press office, please notify them about your upcoming paper to help maximize its impact. If they’ll be preparing press materials, please inform our press team as soon as possible -- no later than 48 hours after receiving the formal acceptance. Your manuscript will remain under strict press embargo until 2 pm Eastern Time on the date of publication. For more information, please contact onepress@plos.org.

Kind regards,

Ahmed Mancy Mosa, Ph.D.

Academic Editor

PLOS ONE

Additional Editor Comments (optional):

Reviewers' comments:

Reviewer's Responses to Questions

**Comments to the Author**

1. If the authors have adequately addressed your comments raised in a previous round of review and you feel that this manuscript is now acceptable for publication, you may indicate that here to bypass the “Comments to the Author” section, enter your conflict of interest statement in the “Confidential to Editor” section, and submit your "Accept" recommendation.

Reviewer #1: All comments have been addressed

**********

2. Is the manuscript technically sound, and do the data support the conclusions?

The manuscript must describe a technically sound piece of scientific research with data that supports the conclusions. Experiments must have been conducted rigorously, with appropriate controls, replication, and sample sizes. The conclusions must be drawn appropriately based on the data presented. 

Reviewer #1: Yes

**********

3. Has the statistical analysis been performed appropriately and rigorously? 

Reviewer #1: N/A

**********

4. Have the authors made all data underlying the findings in their manuscript fully available?

The PLOS Data policy requires authors to make all data underlying the findings described in their manuscript fully available without restriction, with rare exception (please refer to the Data Availability Statement in the manuscript PDF file). The data should be provided as part of the manuscript or its supporting information, or deposited to a public repository. For example, in addition to summary statistics, the data points behind means, medians and variance measures should be available. If there are restrictions on publicly sharing data—e.g. participant privacy or use of data from a third party—those must be specified.

Reviewer #1: Yes

**********

5. Is the manuscript presented in an intelligible fashion and written in standard English?

PLOS ONE does not copyedit accepted manuscripts, so the language in submitted articles must be clear, correct, and unambiguous. Any typographical or grammatical errors should be corrected at revision, so please note any specific errors here.

Reviewer #1: Yes

**********

6. Review Comments to the Author

Please use the space provided to explain your answers to the questions above. You may also include additional comments for the author, including concerns about dual publication, research ethics, or publication ethics. (Please upload your review as an attachment if it exceeds 20,000 characters)

Reviewer #1: It was a pleasure to read because the authors provided sufficient information about their study's major findings and limitations. The manuscript deserves to be published in PLos One because it will aid researchers in performing rapid genetic analysis and hypothesis testing, regardless of programming experience or technical background.

**********

7. PLOS authors have the option to publish the peer review history of their article (what does this mean?). If published, this will include your full peer review and any attached files.

If you choose “no”, your identity will remain anonymous but your review may still be made public.

**Do you want your identity to be public for this peer review?** For information about this choice, including consent withdrawal, please see our Privacy Policy.

Reviewer #1: No

# Editor report: Acceptance letter

**DOI:** 10.1371/journal.pone.0259327.r006

**Authors:** Ahmed Mancy Mosa

27 Apr 2022

PONE-D-21-32657R2 

*EZTraits:* a Programmable Tool to Evaluate Multi-site Deterministic Traits 

Dear Dr. Grabherr:

I'm pleased to inform you that your manuscript has been deemed suitable for publication in PLOS ONE. Congratulations! Your manuscript is now with our production department. 

If your institution or institutions have a press office, please let them know about your upcoming paper now to help maximize its impact. If they'll be preparing press materials, please inform our press team within the next 48 hours. Your manuscript will remain under strict press embargo until 2 pm Eastern Time on the date of publication. For more information please contact onepress@plos.org.

If we can help with anything else, please email us at plosone@plos.org. 

Thank you for submitting your work to PLOS ONE and supporting open access. 

Kind regards, 

PLOS ONE Editorial Office Staff

on behalf of

Dr. Ahmed Mancy Mosa 

Academic Editor

PLOS ONE
